# Prevalence of obstructive sleep apnoea in REM behaviour disorder: response to continuous positive airway pressure therapy

**DOI:** 10.1007/s11325-017-1563-9

**Published:** 2017-09-26

**Authors:** A. Gabryelska, A. Roguski, G. Simpson, E. L. Maschauer, I. Morrison, Renata L. Riha

**Affiliations:** 1 Department of Sleep Medicine, Royal Infirmary Edinburgh, 51 Little France Crescent, Little France, EH16 4SA UK; 2 Department of Sleep Medicine and Metabolic Disorders, Medical University in Lodz, Mazowiecka 6/8, 92-215 Lodz, Poland; 3 Department of Neurology, University of Dundee, Ninewells Hospital, Dundee, DD1 9SY Scotland

**Keywords:** REM behaviour disorder, Sleep apnoea, Sleep-disordered breathing, Continuous positive airway pressure

## Abstract

**Objectives:**

Rapid eye movement behaviour disorder (RBD) is a parasomnia in which there is loss of muscle atonia during rapid eye movement (REM) sleep, resulting in dream enactment. The aims of this study were to determine the prevalence of obstructive sleep apnoea (OSA) in RBD patients and determine whether continuous positive airway pressure (CPAP) therapy improved RBD symptoms in patients with concomitant RBD and OSA.

**Methods:**

A questionnaire was mailed to 120 patients identified from a tertiary sleep centre with RBD meeting full International Classification for Sleep Disorders-3 (ICSD-3) criteria. Patients were diagnosed as having OSA if they had an apnoea-hypopnea index (AHI) ≥ 5. The questionnaire focused on CPAP-use, compliance and complications. Standard statistical analysis was undertaken using SPSS (v.21, IBM).

**Results:**

One hundred and seven of the potential participants (89.2%) had an OSA diagnosis. Out of 72 who responded to the questionnaire, (60%) 27 patients were using CPAP therapy. CPAP therapy improved RBD symptoms in 45.8% of this group. Despite this positive response to treatment in nearly half of CPAP-users, there was no significant difference in subjective or objective CPAP compliance between those who reported RBD improvement and those who did not. Subjective compliance with CPAP was over-reported, with mean usage being 7.17 ± 1.7 h per night compared to objective mean compliance of 5.71 ± 1.7.

**Conclusions:**

OSA is a very common co-morbidity of RBD. CPAP therapy might improve self-reported RBD symptoms further, in addition to standard RBD treatment. However, further research into its topic is necessary.

**Electronic supplementary material:**

The online version of this article (10.1007/s11325-017-1563-9) contains supplementary material, which is available to authorized users.

## Introduction

Rapid eye movement (REM) behaviour disorder (RBD) is a parasomnia characterised by loss of muscle atonia during REM sleep, resulting in excess motor activity [1] and dream enactment (e.g. kicking, punching, shouting, falling out of bed) [2]. These behaviours lead to interrupted sleep as well as injury to the person and/or their bed partner [3]. Poor sleep quality can create an adverse impact on performance at work, and the violent behaviours during sleep can negatively affect relationships.

The prevalence of this condition is estimated to be up to 2% in the elderly population, and even higher in individuals with neuropathological disorders such as Parkinson’s disease (PD), dementia, oligopontocerebellar degeneration and subarachnoid haemorrhage [4, 5]. RBD is most common in males over the age of 50 years [3] and requires overnight video polysomnography (vPSG) to confirm diagnosis as well as exclude other sleep disorders such as obstructive sleep apnoea (OSA) [6].

There are two forms of RBD; idiopathic or secondary to neurological pathology such as narcolepsy, multiple sclerosis, brain injury or neurodegeneration. RBD is also recognised as a side effect of certain medications, such as SSRIs, and has been associated with post-traumatic stress disorder [3]. “Dream-enacting” behaviours are also known to occur in patients with severe OSA [2, 7]. Almost two-thirds of patients originally diagnosed with idiopathic RBD develop dementia and/or PD later in life; therefore, correct diagnosis of RBD is essential to ensure appropriate monitoring, intervention and treatment [2, 8].

Currently, the treatments of choice for RBD are clonazepam and melatonin. However, clonazepam should be used cautiously in patients with concomitant OSA, dementia and gait disorders due to side effects which include early morning motor incoordination, memory dysfunction and increased risk of falling and decreased respiratory tone [9]. Melatonin has been shown to be beneficial in some patients, either as single therapy or as an add-on to clonazepam due to minimal side-effects, no tolerance, dependency, nor hangover effect the following morning [10, 11].

OSA is the cessation of airflow during sleep for at least 10 s accompanied by continued effort to overcome upper airway obstruction [2, 4, 12]. Typical symptoms of OSA include excessive daytime sleepiness, unrefreshing sleep and snoring. OSA has been associated with obesity [13], hypertension [14], cardiovascular events, morbidity, stroke [15, 16] and traffic accidents [17]. The prevalence of OSA has increased considerably in so-called first world countries. Mild (15 < AHI ≥ 5) OSA is estimated to affect up to 34.1% men and 37.4% women, whereas moderate to severe OSA (AHI ≥ 15) affects 49.7 and 23.4% of men and women, respectively [18]. Moderate to severe OSA is usually treated with continuous positive airway pressure (CPAP) which splints the upper airway open, thereby eliminating hypoxia and reducing sleep fragmentation [12].

Despite a plethora of research into RBD and OSA as distinct disorders, there is little literature on the coexistence of RBD and OSA. One study reported a number of patients with OSA experiencing similar behaviours to those seen in RBD at the termination of apnoeic events, suggesting that OSA can sometimes mimic RBD. These patients’ RBD-like symptoms were abolished once CPAP was commenced as treatment for their OSA [2]. A second study found that apnoeas appeared to be less severe in patients with concomitant RBD. The authors postulated that this could have been due to enhanced EMG tone in REM, resulting in increased pharyngeal tone, thereby reducing the number of apnoeas and hypopnoeas in this stage of sleep [4].

The aim of this study was to determine the prevalence of OSA in patients with RBD. We hypothesised that CPAP treatment would improve self-reported RBD symptoms in patients with concomitant RBD and OSA.

## Methods

A questionnaire-based study was undertaken in 120 RBD patients diagnosed and treated at a single tertiary sleep centre between the years 2000–2016. Potential participants were recruited from an existing cohort of RBD patients diagnosed at the Department of Sleep Medicine, Royal Infirmary of Edinburgh.

All patients were sent an anonymised questionnaire with a cover letter to explain the study. A self-addressed stamped return envelope was also enclosed. A second mail-out was undertaken 4 weeks later to those who had not returned the questionnaire, in order to enrich the return rate.

The questionnaire was designed to focus on areas previously described, validated and published by White et al. [19] with special emphasis placed on CPAP use including compliance and complications.

All patients included in this study had been assessed and investigated clinically by the authors (IM, RLR) and diagnosed in accordance with either the ICSD-2 or ICSD-3 criteria [20]. Overnight video PSG (vPSG), scored according to criteria, used by Montplaisir el al. [21] with additional self-report of dream re-enactment was used to confirm the diagnosis of REM sleep without atonia (RSWA). Inclusion in this study required phasic chin EMG signal during REM sleep at least twice the background activity or greater than 10 μV, for 50% or more of the duration of the epoch. Phasic EMG density was defined as the percentage of 2 s mini epochs with phasic twitches, with the amplitude more than four times that of the background EMG activity. Patients were excluded from the study if their excess EMG activity was concurrent with respiratory events and snoring. Patients were also excluded if they had a diagnosis of concurrent epilepsy, sleep-related movement disorders or arousal disorders. Diagnosis of concomitant OSA was confirmed with either an inpatient nocturnal vPSG study or with a home study enlisting a portable monitoring device. Time of diagnosis of both conditions varied in different patients. In many cases, OSA and RBD diagnosis were made at the same time during a vPSG. However, in some individuals, RBD and OSA developed independently of each other and were diagnosed using two separate studies.

Ethical approval was not deemed necessary by the local research committee, and consent to participate was implied if the patients returned the questionnaire. All research was carried out in accordance with the Helsinki criteria [22].

Statistical analysis was undertaken using SPSS (v. 21 IBM). The chi-square test and Fisher’s exact test were used for discrete variables. The Student *t* test and Mann-Whitney *U* test were used for continuous variables. Results are reported as mean ± standard deviation for normally distributed data and median with interquartile range (IQR 25–75%) for non-parametrically distributed data. Statistical significance was taken at *p* < 0.05, and all tests were two-tailed.

## Results

Of the 120 patients included in the study, 102 (85%) were male and 18 (15%) were female. The mean age of the subjects at the time of the study was 57 ± 15 years with an age range of 23 to 85 years (the 23-year-old man had a concurrent diagnosis of post-traumatic stress disorder (PTSD)).

Seventy-two (60%) patients responded to the questionnaire; 61 (84.7%) males and 11 (15.3%) females. Of the 48 non-responders, 41 (85.4%) were male and 7 (14.6%) were female. Of those who did not respond, one was deceased and ten had an incorrect address. Patients were significantly more likely to reply to the questionnaire if they were married or had a partner (*p* < 0.0001) and if they were over the age of 50 years (*p* < 0.0001). Mean age of responders was 61.4 ± 12.2 years compared to non-responders, who had a mean age of 50.69 ± 16.6 years (*p* < 0.0001).

For the purpose of this study, patients were defined as having OSA meeting treatment guidelines (SIGN) if they had an AHI of ≥ 5 per hour [18]. We further classified participants as mild/moderate OSA (AHI ≥5 < 15) and moderate-severe OSA (AHI ≥ 15) for detailed analysis. Table 1 shows characteristics of patients with and without OSA in the group of RBD patients as a whole. Patients with concomitant RBD and OSA were significantly more likely to be male (*p* = 0.004).

**Table 1:** Characteristics of RBD + OSA (AHI ≥ 5 ≤ 15), RBD + OSA (AHI ≥ 15) and RBD without OSA patients (*n* = 120)

	RBD + OSA (AHI ≥ 5 < 15)	RBD + OSA (AHI ≥ 15)	RBD without OSA	*p* value
M:F ratio	30:12	62:3	10:3	0.004
ESS (*n*/24)	7.32 ± 5.23	9.07 ± 5.38	9.54 ± 7.68	0.25
BMI (kg/m^2^)	28.89 ± 6.83	30.63 ± 6.11	27.12 ± 2.86	0.12
Age at diagnosis	51.71 ± 15.8	56.24 ± 13.36	50.1 ± 16	0.2
AHI/h	10.55 ± 2.74	36.11 ± 22.9	3.62 ± 1.1	< 0.0001
Percentage of the sample	35	54.2	10.8	–

Characteristics of patients with and without OSA in the group of RBD patients as a whole

### CPAP treatment

Twenty-seven of the 72 patients (37.5%) who responded to the questionnaire were using CPAP. None of the responders had been referred for surgery or construction of a mandibular repositioning device for their OSA. Most users of CPAP were male (25 men vs. 2 women) and 11 out of 24 patients (45.8%—response not given by three participants) reported improvement in their RBD symptoms as a result.

Table 2 shows data for AHI, self-reported average hours of use of CPAP, Epworth sleepiness scale (ESS) at outset of treatment and RBD improvement with CPAP. Apart from AHI at baseline, there was no significant difference between the groups.

**Table 2:** Characteristics of CPAP-users vs. non-CPAP users in questionnaire responders

	CPAP-user (*n* = 27)	Non-CPAP user (*n* = 45)	*p* value
Male (*n*)	25 (92.6%)	36 (80%)	0.15
Age (years)	57.37 ± 10.78	57.75 ± 12.87	0.89
BMI (kg/m^2^)	30.24 ± 24.89	28.65 ± 4.61	0.16
ESS (*n*/24)	8.52 ± 4.88	6.59 ± 5.72	0.16
AHI/h	30.24 ± 24.89	16.78 ± 16.06	0.02
Live with partner (*n*)	23 (85.2%)	37 (82.2%)	0.7
Self-reported CPAP usage (hours)	7.17 ± 1.7	–	–
*Objective CPAP usage (hours)*	5.71 ± 1.7		
RBD improvement with CPAP	11/27 (45.8%)	–	–

Data for AHI, hours of CPAP use, Epworth Sleepiness Score (ESS) at outset of treatment and RBD improvement with CPAP

Average hours of self-reported use of CPAP per night were 7.17 ± 1.7 h (*n* = 18) compared to objective CPAP use per night of 5.71 ± 1.7 h (*n* = 15). The majority of responders were married or in a relationship. Over half of patients treated with CPAP experienced difficulties when using the device including the following: noisiness (*n* = 1), air leak/poor fit (*n* = 8), discomfort (*n* = 1), dry throat (*n* = 1), a suffocating feeling (*n* = 1), facial sores (*n* = 2) and cuts on the nose (*n* = 2).

Almost half (45.8%) of the CPAP users noted an improvement in their RBD symptoms with the use of the CPAP machine. There was no significant difference in self-reported or objective CPAP compliance between individuals who reported RBD improvement and those who did not. Self-reported CPAP compliance was 6.72 ± 1.48 h/night in individuals who experienced improved RBD symptoms, compared to 7.58 ± 2 h/night for those who did not (*p* = 0.32). For objective compliance, mean hours/night were 5.16 ± 1.52 compared to 5.7 ± 3.43, respectively (*p* = 0.73). OSA severity (mild/moderate vs. moderate-severe) also did not influence RBD response to CPAP (*p* = 0.42). Individuals reporting improved RBD on CPAP demonstrated a non-significantly higher ESS at baseline than those who did not experience RBD improvement (10.5 ± 5.16 vs. 7.23 ± 4.24, *p* = 0.11), suggestive of a worse baseline sleep quality.

### Medications

Of the 72 responders, 38 (54.3%) were taking clonazepam or melatonin for their RBD at the time of the study. Thirty-five (87.5%) of these patients were married or living with a partner. Thirty-one patients were taking clonazepam alone, and two were taking melatonin alone, with further five patients taking both medications together. Average dose of each treatment and the response to treatment is shown in Table 3.

**Table 3:** Medication use for RBD in questionnaire responders (*n* = 72)

	CPAP-user	Non-CPAP user	*p* value
*Clonazepam* (*n* = 31)	6	24	0.05
Dose clonazepam (mg)	2.32 ± 1.77	1.36 ± 1.24	0.16
RBD improvement with clonazepam	5/6	19/24	1.0
*Melatonin* (*n* = 2)	1	1	0.44
Dose melatonin (mg)	4.25 ± 2.06	2.33 ± 0.57	0.3
RBD improvement with melatonin	1/1	1/1	1.0
*Clonazepam + melatonin* (*n* = 5)	3	2	0.3
RBD improvement with clonazepam + melatonin	3/3	2/2	1

Average dose of each treatment medication and the response to treatment

Self-reported compliance with medication was not significantly different (*p* = 0.74) in those using CPAP compared to non-users of CPAP. Of the 11 CPAP-users who answered this question, ten took their medication 7 days a week (90.9%), compared to 23 of 26 non-CPAP users (88.5%). Reasons for not taking medication everyday included the following: not wanting to depend on medication, symptoms reducing in frequency or severity and being advised by their GP not to take the medication(s) everyday. Thirty-two of 34 (94%) patients with a spouse/partner who responded to the question took their medication 7 days per week (*p* < 0.0001). Of the 12 patients who returned the questionnaire and were single, only four responded to the question, all stating that they complied 7 days a week.

### Lifestyle changes

Fifteen CPAP users were advised to make lifestyle changes by their doctor, whereas 17 patients who did not use CPAP were advised to make lifestyle changes. Lifestyle changes suggested ranged from reducing alcohol and caffeine intake to using a bed guard and increasing exercise. Half (50%) of the responding CPAP users (*n* = 7) and nine of the responders not using CPAP (52.9%) noticed an improvement (reduction in frequency and severity) in their RBD symptoms after making these lifestyle changes. The majority of patients (52%, *n* = 39) were not given specific lifestyle recommendations. Caffeine reduction was the most commonly suggested change (22.7%).

## Discussion

This is the first study to specifically explore the effects of treating sleep disordered breathing (SDB) in RBD. There have been few papers previously published discussing concomitant RBD and OSA. Response to the questionnaire was excellent, with 60% of patients responding. Of 120 patients, 89.2% had RBD plus an AHI ≥ 5/h suggesting that RBD/OSA is common, particularly in older populations, irrespective of BMI. Patients with concomitant RBD and OSA were significantly more likely to be male (*p* = 0.004). A study from the USA showed OSA prevalence in RBD to be 34% [23], and another case series from Hong Kong reported the prevalence to be 61% [24]. Our study results are similar to these figures. However, the prevalence of OSA in RBD may have been underestimated as these aforementioned studies used an AHI ≥ 10/h to diagnose OSA and this study used AHI ≥ 15. If a cutoff of AHI ≥ 10/h is applied to our study, the prevalence amongst our responding RBD cohort rises to 75% (*n* = 120).

Patients in this study reported relatively high compliance with CPAP treatment compared to other studies [4, 25], with average use of 7.17 ± 1.7 and 5.71 ± 1.7 h per night in subjective and objective assessment, respectively. All patients attended a tertiary sleep centre, and it is possible that they may have been exposed to multiple treatment regimens before and therefore may have understood the benefits of treatment better. Studies have shown those who adhere to one treatment are more likely to adhere to others [26]. The patients in this study reported regular use of their current RBD medications; this medication compliance is reflected in the high-CPAP compliance that was demonstrated objectively. The results also point to the variability of RBD and its expression.

Almost half (45.8%) of patients on CPAP noticed an improvement in their RBD symptoms with CPAP treatment supporting our hypothesis that CPAP may improve RBD concomitant with OSA. However, there was no significant difference in the subjective or objective compliance of individuals who experienced RBD improvement on CPAP therapy compared to those who did not. This suggests that degree of CPAP compliance does not affect severity of RBD symptoms.

Iranzo et al. reported that severe OSA can imitate RBD symptoms and that this phenomenon can be successfully treated by CPAP [2]. This suggests true RBD could be exacerbated by OSA. With most of the RBD behaviours occurring at the termination of apnoeic events [2], if the apnoeas in those with OSA are treated, then the abnormal dream enactment behaviours associated with RBD may not occur or may occur with less severity or frequency. The RBD symptom improvements reported by approximately half of the responders may therefore have been due to a reduction in pseudo-RBD behaviours associated with concomitant OSA.

Another reason CPAP may benefit RBD in patients with concomitant OSA is by reducing sleep fragmentation [12]. Theoretically, OSA could be causing pseudo-RBD symptoms in addition to true RBD or OSA could be exacerbating RBD through sleep fragmentation, arousals and sleep disruption [2]. It is therefore feasible that patients using CPAP would report a decrease in RBD events, as they are unable to distinguish which of their symptoms are caused by RBD and which symptoms are the result of OSA-related pseudo-RBD. No study has ever investigated spectral densities of REM EEG in RBD, but there is evidence that REM EEG activity is decreased in OSA [27] just as there is evidence supporting changes in EEG density in slow-wave sleep disorders [27].

Interestingly, CPAP compliance was not statistically different between individuals with concomitant OSA who reported improved RBD with CPAP therapy and those who did not. This suggests that the extent of CPAP use does not affect RBD symptom severity, and therefore, other factors must be considered which may positively influence RBD expression. While we report that OSA severity (mild/moderate vs. severe) does not affect RBD response to CPAP therapy, the OSA phenotype may influence treatment response. For example, individuals who suffer from REM-dependent OSA (apnoeas and hypopnoeas present during REM sleep) [28] may experience a subjective improvement in RBD symptoms on CPAP treatment compared to individuals with position-dependent OSA (apnoeas and hypopnoeas present in supine position). REM-dependent OSA results in disturbed REM sleep, which in turn might exacerbate RBD; therefore, improved REM sleep with CPAP use might act to decrease RBD behaviours. There are no reports in the literature exploring this relationship between OSA phenotype, RBD and CPAP-response, demonstrating a need for further research.

CPAP therapy can create REM rebound when treatment commences [5], ultimately increasing the number of RBD episodes experienced by the patient. Although this phenomenon is generally short-lived, it is important that patients are informed of this potential side effect to ensure they persevere with CPAP treatment.

The majority of patients diagnosed with RBD are prescribed pharmacological treatment to treat the disorder. In our study, over half of patients were currently taking either one of or a combination of clonazepam and melatonin. Due to the respiratory-depressive effects of benzodiazepines on muscle tone [29], clonazepam may aggravate symptoms of OSA, subsequently increasing a patient’s AHI. An increase in OSA symptoms may cause a secondary increase in RBD symptoms due to sleep fragmentation.

One of the limitations of this study was the use of a self-reporting questionnaire with its attendant biases. For future studies, it would be beneficial to create a standard instrument from existing questionnaires to be used longitudinally during a set period of time from CPAP commencement. Patients responding to our questionnaire tended to be older with a spouse/partner. This may also have increased the likelihood of adherence to therapy, as it has been shown that marital status contributes to therapy adherence [30, 31]. Similarly, individuals who experienced a positive response to CPAP may have been more likely to respond to the questionnaire than those who found no benefit.

We explored the effect of three treatment options on RBD symptoms. Many of the individuals in our cohort were on several treatments and may have been unable to determine which improved their RBD. Further investigation between the interactions of different RBD treatments is required.

The study sample size, though small, is large for a reasonably uncommon condition such as RBD. In the future, a follow-up vPSG study for all patients, in order to objectively quantify changes in RSWA, would be ideal. This would eliminate the inevitable recall bias of questionnaire methodology regarding improvement in RBD symptoms, especially given the 17-year recall period.

The level of detail regarding changes to RBD symptoms was limited by the phrasing of questions within the questionnaire. Therefore, data regarding worsening of RBD symptoms and the nature of RBD improvement (for example, severity or frequency of RBD behaviours) was not available for analysis and the potential disadvantages of CPAP use in RBD patients cannot be further explored in this study.

The high prevalence of concomitant OSA in RBD establishes that it occurs commonly. CPAP therapy is safe and effective in the context of RBD and sleep-disordered breathing and may enhance the effects of (and therefore reduce) medication requirements in these patients. If OSA is significant or contributing to additional nocturnal symptoms such as snoring and daytime sleepiness, CPAP can be trialled safely. Patients diagnosed with concomitant RBD and OSA should be given the opportunity to undergo a trial CPAP if clinically indicated. This may improve overall sleep quality and health. However, further research in this area is necessary in order to establish any clear effects of CPAP treatment on RBD per se.

## Electronic supplementary material

ESM 1 (DOC 95 kb)
