# Supplementary material for: Prevalence of obstructive sleep apnoea in REM behaviour disorder: response to continuous positive airway pressure therapy
Source: Sleep Breath. 2017 Sep 26;22(3):825–30. doi: 10.1007/s11325-017-1563-9 (PMC6133117; doi:10.1007/s11325-017-1563-9)
Supplement: Supplementary file 1 — (DOC 95 kb) [file 11325_2017_1563_MOESM1_ESM.doc]

*Dear_________________________,*

*Enclosed is a short questionnaire on REM behaviour disorder which is part of a larger audit that we are currently undertaking in the Department of Sleep Medicine at the Royal Infirmary of Edinburgh to assess our quality of care for patients like yourself.*

*Thank you for taking the time to fill out this quick questionnaire.*

*The purpose is to gather information on REM behaviour disorder (RBD), in particular the events leading up to diagnosis and the treatment of the disorder.*

*All answers will be treated in the strictest confidence and are being used for the purposes of audit only.*

*Please return the completed questionnaire in the self-addressed, stamped envelope enclosed.*

*If you have any questions of concerns regarding this questionnaire please do not hesitate to contact us on the numbers above.*

*Thank you again.*

*Kind regards,*

*Renata L Riha Gillian Simpson Ian Morrison*

*Consultant Sleep Medicine Medical Student Consultant Neurology*

Your current age: Male/Female:

Marital Status: Occupation:

1. Which year was your REM behaviour disorder diagnosed?
2. Which year, approximately, did your symptoms begin?
3. Had you heard of REM behaviour disorder prior to your diagnosis? YES / NO
4. Does anyone in your family have REM behaviour disorder?

YES / NO

1. Do you feel you have a good understanding of REM behaviour disorder? YES / NO

Have the following been useful in helping your understanding? (please circle)

*Information from doctor TV Internet*

*Other _________________*

Is there any resource you think would improve your understanding?

1. If there is a delay between 1 and 2, (see above), is there a reason for this delay? (tick as appropriate)

| Sleep behaviour initially mild/infrequent |  |
| --- | --- |
| Felt too busy to consult a doctor |  |
| Felt too healthy to consult a doctor |  |
| Did not think it was serious enough to see doctor |  |
| Thought this was “normal” sleep behaviour |  |
| Thought the sleep behaviour would eventually settle |  |
| Did not realise there were treatment options |  |
| Was unaware of behaviour (no bed partner/work abroad) |  |
| Embarrassed to discuss behaviour |  |
| Doctor did not recognise RBD (REM behaviour disorder) |  |
| Was never asked about sleep by doctor |  |
| RBD was initially diagnosed as another condition |  |
| Other – please state |  |

1. Did you go to the doctor specifically about your sleep behaviour?

YES / NO

1. Was the decision to consult the doctor made by (tick as appropriate):

| You |  |
| --- | --- |
| Your partner |  |
| Both you and your partner |  |
| Other (please state) |  |

1. How did these factors contribute to your decision to consult a doctor?

|  | No impact | | Small impact | Moderate  impact | Strong impact | Very strong |
| --- | --- | --- | --- | --- | --- | --- |
| Injury to self |  | |  |  |  |  |
| Injury to partner |  |  | |  |  |  |
| Increased frequency of behaviour |  |  | |  |  |  |
| Increasing injury/violence |  |  | |  |  |  |
| Partner noticed more regular behaviour |  |  | |  |  |  |
| Partner noticed increase in **violence** of behaviour |  |  | |  |  |  |
| Partner moved into separate bed |  |  | |  |  |  |
| Impact on job |  |  | |  |  |  |
| Impact on relationship |  |  | |  |  |  |
| Other (please state) |  |  | |  |  |  |

1. Did the symptoms occur at the same time as taking certain medication? YES / NO

If **YES** please state which medication:

1. Did your REM behaviour disorder start at the same time as a stressful life event (for example, new job, new home, bereavement)? YES / NO

If **YES,** please state the event:

1. Does anything trigger your REM behaviour disorder or makes it occur more regularly or with increased force/violence?

YES / NO

If **YES** please describe:

1. Do any of the following themes occur in your dreams during episodes of REM behaviour disorder? (tick if appropriate)

| Job/ previous job |  | Violence |  |
| --- | --- | --- | --- |
| Relationship |  | Stressful events currently in your life |  |
| Other (please state) |  |  |  |

1. If you are FEMALE:

Does your RBD change throughout your menstrual cycle? YES / NO

If **YES**, please describe:

Do you have any children?

If **YES**, how many?

How was your REM behaviour disorder during pregnancy? (please circle)

*Better Worse No change*

1. **What medication are you taking for RBD at present?**

|  | Tick | Dose | Date started |
| --- | --- | --- | --- |
| No treatment |  |  |  |
| Clonazepam |  |  |  |
| Melatonin |  |  |  |
| Other(state) |  |  |  |

1. Do you think this treatment has improved your REM behaviour disorder?

YES / NO

1. On average, how many days per week do you take your medication? (please circle)

*0 1 2 3 4 5 6 7 varies*

1. If you do not take your medication every day, please state why:
2. Do you experience any side effects from the above treatment(s)?

YES / NO

If **YES,** please state:

1. Have you taken any of these medications **in the past** for your RBD?

| Treatment | Tick | Reason for stopping |
| --- | --- | --- |
| Clonazepam |  |  |
| Melatonin |  |  |
| Other  (please state) |  |  |

1. Were you advised to make lifestyle changes? YES / NO

If **YES**, tick all that apply:

| Stress control |  | Increased exercise |  |
| --- | --- | --- | --- |
| Alcohol reduction |  | Caffeine reduction |  |
| Sleeping in separate room to partner |  | Sleeping in separate bed to partner |  |
| Putting a “guard” around bed |  | Other (please state) |  |

1. Do you feel these lifestyle changes made a difference? YES / NO
2. Do you have any of the following sleep problems? (please tick)

| Sleep apnoea |  | Sleep walking |  |
| --- | --- | --- | --- |
| Insomnia |  | Sleep talking |  |
| Restless leg syndrome |  | Other sleep disturbance (please state) |  |
| Narcolepsy |  |  |  |

1. If **YES** to any of the above, are you receiving treatment? YES / NO
2. If **YES** please state what treatment you are receiving:
3. Has this improved your REM behaviour disorder? YES / NO
4. Do you use a CPAP machine at night? YES / NO

If, **YES**:

Has this improved your REM behaviour disorder? YES / NO

How many hours per night do you use CPAP?

Have you experienced any difficulties with CPAP? YES / NO

If YES, please describe:

1. Have you ever had any of the following conditions? (tick any that apply)

| Asthma |  | Emphysema |  | High blood pressure |  | Angina |  |
| --- | --- | --- | --- | --- | --- | --- | --- |
| Stroke |  | Kidney/liver problems |  | Thyroid problems |  | Heart attack |  |
| Depression/ Anxiety |  | Epilepsy |  | Parkinson’s Disease |  | Diabetes |  |

1. Finally, has your sense of smell (tick one that applies best to you):

| Stayed the same over time |  | Improved over time |  |
| --- | --- | --- | --- |
| Diminished over time |  | Never had a sense of smell |  |

*Many thanks for completing this questionnaire.*
